# A Statistical Approach for *A-Posteriori* Deployment of Microclimate Sensors in Museums: A Case Study

**DOI:** 10.3390/s22124547

**Published:** 2022-06-16

**Authors:** Francesca Frasca, Elena Verticchio, Paloma Merello, Manuel Zarzo, Andreas Grinde, Eugenio Fazio, Fernando-Juan García-Diego, Anna Maria Siani

**Affiliations:** 1 Department of Physics, Sapienza University of Rome, P.le A. Moro 5, 00185 Rome, Italy; f.frasca@uniroma1.it; 2 Department of Earth Sciences, Sapienza University of Rome, P.le A. Moro 5, 00185 Rome, Italy; elena.verticchio@uniroma1.it; 3 Department of Accounting, University of Valencia, Av. dels Tarongers s/n, 46022 Valencia, Spain; paloma.merello@uv.es; 4 Department of Applied Statistics, Operations Research and Quality, Universitat Politècnica de València, Camino de Vera s/n, 46022 Valencia, Spain; mazarcas@eio.upv.es; 5 Royal Danish Collections, Øster Voldgade 4A, 1355 Copenhagen, Denmark; ag@kosa.dk; 6 Department of Fundamental and Applied Sciences for Engineering, Sapienza Università di Roma, Via A. Scarpa 16, 00161 Roma, Italy; eugenio.fazio@uniroma1.it; 7 Department of Applied Physics (U.D. Industrial Engineering), Universitat Politècnica de València, 46022 Valencia, Spain; fjgarcid@upvnet.upv.es

**Keywords:** museum, microclimate, multivariate approach, principal component analysis, cluster analysis, sensors, deployment, temperature, relative humidity

## Abstract

The deployment of sensors is the first issue encountered when microclimate monitoring is planned in spaces devoted to the conservation of artworks. Sometimes, the first decision regarding the position of sensors may not be suitable for characterising the microclimate close to climate-sensitive artworks or should be revised in light of new circumstances. This paper fits into this context by proposing a rational approach for a posteriori deployment of microclimate sensors in museums where long-term temperature and relative humidity observations were available (here, the Rosenborg Castle, Copenhagen, Denmark). Different statistical tools such as box-and-whisker plots, principal component analysis (PCA) and cluster analysis (CA) were used to identify microclimate patterns, i.e., similarities of indoor air conditions among rooms. Box-and-whisker plots allowed us to clearly identify one microclimate pattern in two adjoining rooms located in the basement. Multivariate methods (PCA and CA) enabled us to identify further microclimate patterns by grouping not only adjoining rooms but also rooms located on different floors. Based on these outcomes, new configurations about the deployment of sensors were proposed aimed at avoiding redundant sensors and collecting microclimate observations in other sensitive locations of this museum.

## 1. Introduction

The climate surrounding cultural objects (namely the microclimate or indoor climate) can affect their chemo-physical and structural properties over the years. The analysis of microclimate data has become an increasingly common practice to pinpoint the agents causing the deterioration of artworks. In this context, one of the objectives of the European project CollectionCare (www.collectioncare.eu (accessed on 1 April 2022) [1]) is the continuous monitoring of the environmental conditions (e.g., temperature, relative humidity, light, and air pollutants) to which an individual artwork is exposed during exhibition, storage, handling, or transport.

The choice of sampling points in which the sensors should be deployed is a demanding task to perform, as an inappropriate position of sensors could compromise the resulting microclimate characterisation. Indeed, the horizontal and vertical distributions of hygrothermal variables in conservation spaces (e.g., museums, historical buildings, places of worship, archaeological sites) are rarely homogeneous due to several factors (e.g., exposure to solar radiation, wall thickness and insulation, air leakage, glass panes, or mass tourism [2,3]). Therefore, a given sensor placed in a sampling point might not be representative of the whole air surrounding the artwork, possibly leading to a partial evaluation of the climate-induced degradation risks, especially in large spaces [4]. Specific requirements for climate and weather stations are established by WMO [5], although these rules do not always fit in the case of indoor climate monitoring. In the Preventive Conservation (PC) field, the current standards EN 15758:2010 [6] and EN 16242:2012 [7] provide recommendations on the choice of the type of sensor devices to be used, and they suggest locating them as close as possible to climate-sensitive artworks.

### 1.1. Scientific Literature about Deployment of Microclimate Sensors

A common procedure designed for microclimate monitoring in exhibition spaces or storage of artworks has not yet been established in the literature. Generally speaking, if the number of sensors is fixed (e.g., limitation in budgetary availability), the sensor deployment is strongly guided by several conflicting instances, such as the availability of electricity and/or radio/Wi-Fi connection availability, the aesthetic impact, the sensor accessibility to replace batteries or download data, etc. To the best of our knowledge, few papers on microclimate studies in conservation spaces and historical buildings have investigated the issue of the deployment of sensors, i.e., which criteria should be considered for the optimum location of sensors.

Most authors describe the rationale for the sensors’ deployment according to some well-established criteria [3] to avoid radiating sources, air flow through the opening of doors/windows, heat loss through external walls, etc. (to cite a few, [8,9,10]). Actually, some authors set out the deployment of wireless sensors after analysing all the factors involved in restricting signal propagation and coverage [11,12]. More recently, the authors of this paper proposed installed multiple thermo-hygrometers in a library repository after conducting microclimate field campaigns in different time slots to study the horizontal and vertical distributions of temperature and mixing ratio of moist air [13].

In other cases, the identification of the best location for microclimate sensors was conducted to specifically detect peculiar microclimate behaviours or episodes. For example, Merello et al. [14] carried out thermographic research to identify the optimum location of thermo-hygrometers designed to study the vertical gradient of temperature and relative humidity. Visco et al. [15] used the sampling design method to deploy 15 sensors aimed at collecting temperature, relative humidity, and light measurements to characterise the whole indoor space. Aste et al. [16] carried out microclimate field campaigns with 42 sensors deployed in a three-dimensional virtual grid inside the Duomo di Milano (Milan, Italy) and identified the most representative positions for six microclimate sensors, also considering the criterion of easy accessibility by technicians. The visual disturbance of sensors to visitors, instead, was the criterion adopted by Lucero-Gómez et al. [17] to deploy sensors in Gallerie dell’Accademia (Venice, Italy).

When it comes to monitoring valuable and vulnerable surfaces, other criteria should be identified. Lucchi et al. [18] transferred the restoration theories for preserving the identity of artworks into a practical guide to deploy microclimate sensors on artworks. Several non-destructive testing (NDT) techniques, such as infrared thermography (IRT), can be used to support the choice of the most appropriate location of sensors [19].

All the above-mentioned studies dealt with the first-guess (or initial) installation of sensors for microclimate monitoring. Siani et al. [20] (Museo Napoleonico in Rome, Italy) and Ramirez et al. [21] (L’Almoina archaeological site in Valencia, Spain) proposed multivariate statistical methodologies to optimise the number of sensors as a strategic approach to reduce sensor maintenance/calibration costs and redundancy in data analysis.

### 1.2. Research Aims

The main purpose of this paper is to propose a rational approach for a posteriori deployment of microclimate sensors in conservation spaces once at least one year of microclimate data was collected. Indeed, it could happen that in the first-guess configuration, sensors can be deployed without the knowledge of the microclimate behaviour. It follows that some sensors might be exposed to similar microclimate conditions. In such a case, the identification of analogous microclimate patterns can support the optimisation of sensor deployment by avoiding their redundancy. This way, redundant sensors can be employed to collect microclimate data in other rooms ruled out due to budgetary limitations during the first-guess configuration.

To this purpose, three statistical methods at different levels of complexity were applied to provide an objective climate-room classification: one univariate and two multivariate methods. The research goes a step further in the use of multivariate methods for an objective deployment of sensors. Although the three statistical methods are commonly used individually, this paper represents the first attempt to compare their capability in real world by also providing pro and cons. The approach was tested in Rosenborg Castle (Copenhagen, Denmark), which is one of the museum partners of the European project CollectionCare, where microclimate measurements have been recorded in 14 rooms since 2012.

Section 2 is devoted to the description of the case study, the collection of microclimate data, and the statistical methodology adopted to test the approach. The main outcomes are presented and discussed in Section 3. Finally, the effectiveness of the proposed approach is summarised in Section 4.

## 2. Materials and Methods

### 2.1. The Case Study: Rosenborg Castle

**Figure 1:**
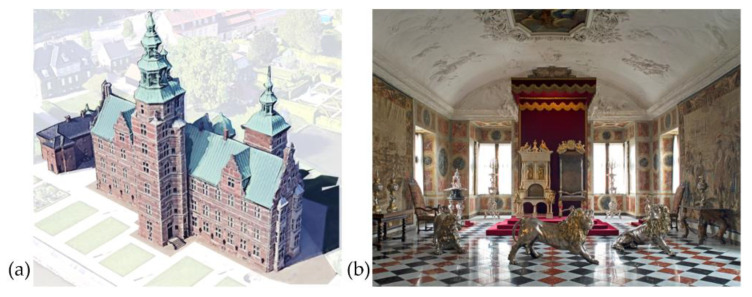
Rosenborg Castle: (**a**) 3D sketch of the external view; (**b**) the Great Hall (Room 21) located on the second floor.

Rosenborg Castle (Figure 1a) is a Renaissance castle located in Copenhagen, Denmark (Lat. 55.7° N and Long. 12.6° E, height 7 m a.m.s.l.). The Kings Collection is housed in 27 rooms and consists of artistic artefacts, paintings, and tapestries illustrating the culture and art of the Danish kings from the 17th to the 19th century (Figure 1b). The building has four floors including the basement, an attic, and three towers, with an area per floor of about 350 m^2^. The external walls are about 1.0 m thick, except in the basement with a thickness of 1.3 m. Windows are single pane, and most rooms are equipped with cast iron radiators that are turned on in cold periods.

Measurements of air temperature (T) and relative humidity (RH) have been systematically recorded in fourteen exhibition rooms since 2012. The deployment of the existing sensors was decided by museum conservators over time, based on different criteria and specific conservation needs in particular rooms, but without a collective plan. This study used indoor T and RH observations from October 2012 to December 2018, collected by thermo-hygrometers (Tinytag View 2 TV-4050 from Gemini Data Loggers Ltd. (Chichester, UK)), hereafter cited as TRH. Data were downloaded on-site every 12 months (Table 1), when the batteries of the sensors were also replaced. These thermo-hygrometers consisted of a thermistor for T measurements (uncertainty 0.4 °C in the range 0–50 °C) and a film capacitive sensor for RH measurements (uncertainty 3.0% in the range 0–100%), with features in accordance with the requirements recommended by the European standards [6,7].

**Figure 2:**
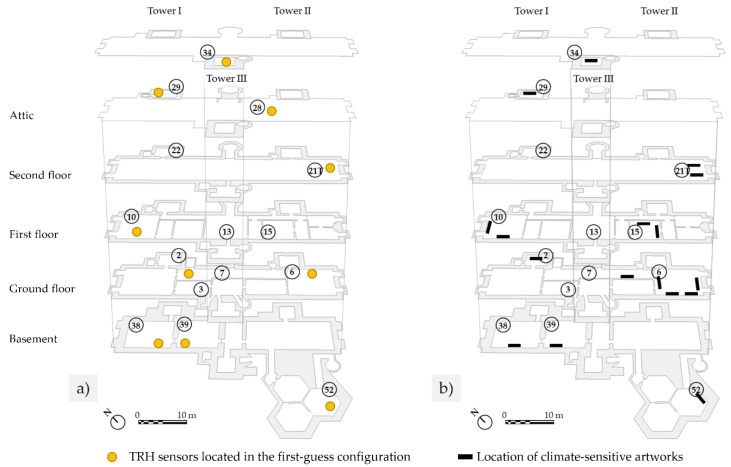
Plans of Rosenborg Castle: (**a**) First-guess configuration of the TRH sensors (yellow circles). (**b**) Location of those artworks (black parallelepipeds) which were selected by museum conservators in the framework of the CollectionCare project. Room numbers are provided in the circles and also correspond to the associated TRH sensor.

The sampling rate chosen was 60 min, which is considered appropriate for microclimate investigations in museums [22]. Each TRH was named according to the room number where it was located, at a height ranging from 0.40 to 2.00 m (average value of 1.8 m); this deployment was named “first-guest configuration” (Figure 2a). When possible, the sensors were put behind a barrier or on top of a piece of furniture/facing away from the audience to prevent them from being moved. No sensors were located in Tower I and II, while two data loggers were placed in Room 21, coded as 21 in the north side and 21T, placed close to the throne in the south-east corner.

Figure 2b shows the location of artworks selected by conservators on the basis of their climate vulnerability in the framework of the CollectionCare project.

### 2.2. Microclimate Statistical Data Analysis

**Figure 3:**
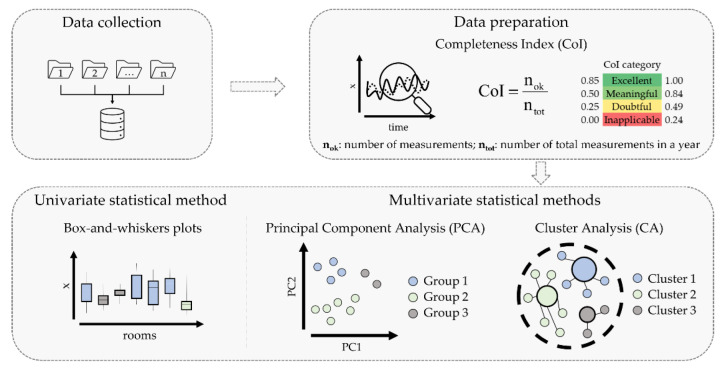
Schematic workflow of the approach: Data collection from several sensors located in different rooms; data pre-processing to select long-term time series in multi-room observations; application of statistical methods to identify microclimate similarities among rooms to be associated with climate-room blocks.

The analysis entails a two-step procedure after data collection, as schematised in Figure 3:

1. Data preparation (Section 2.2.1): selection of a continuous long-term TRH time series (at least one calendar year).
2. Application of the statistical methods:
  - univariate statistics (Section 2.2.2): box-and-whisker plots;
  - multivariate statistics (Section 2.2.3): cluster analysis (CA) and principal component analysis (PCA).

These statistical methods were chosen as they were already used for the characterisation of microclimate behaviour in other cultural/historical sites and proved to be effective for an objective identification of microclimate similarities among rooms (if any) that might be associated with climate-room patterns.

All the analyses were performed with MatLab R2020a (box-and-whisker plots and Cluster Analysis) and Stata 14 software (Principal Component Analysis).

This approach was intended to develop a posteriori deployments of microclimate sensors (Section 3.4). In this phase, we assumed that the number of sensors to relocate was equal to the number of sensors that collected similar microclimate patterns (i.e., redundant sensors).

The proposed relocation of such sensors followed the priority provided by conservators, i.e., considering the climate-sensitivity of the selected artworks.

#### 2.2.1. Data Preparation

The Completeness Index (CoI) was used to establish a period in which there is at least one year of continuous observations with a reduced number of missing values in each of the 14 rooms. The CoI is defined as the ratio between the number of measurements collected and the total number of recordings in one year [23]. This index ranges between 0 (no measurements, all data discarded for instrumental problems) and 1 (no missing values). Depending on the CoI value, the quality of the microclimate data series for the application of any further analysis was classified as: excellent CoI = [0.85, 1.00], meaningful CoI = [0.50, 0.84], doubtful CoI = [0.25, 0.49], or inapplicable CoI = [0.00, 0.24]. In this work, the CoI was iteratively calculated from October 2012 until December 2018 for T and RH observations collected in each room. A period of 12 consecutive months was used here, not necessarily from January until December. A value of CoI > 0.85 was established as a criterion so that only less than 15% of data was missing. This way, both long- and short-term variability of T and RH data could be adequately studied to characterise the microclimate within exhibition rooms.

#### 2.2.2. Univariate Statistical Method

*Box-and-whisker plots*. For each of the selected exhibition rooms, the microclimate was first investigated through box-and-whisker plots in order to visualise and compare TRH values collected in several rooms and to identify similarities among them. Outdoor T and RH data were also plotted to assess their influence on indoor conditions. Outdoor T and RH data in Copenhagen were retrieved from the fifth generation ECMWF (European Centre for Medium-Range Weather Forecast) reanalysis—hereafter called ERA5—via the Climate Data Store (CDS) infrastructure [24]. Box-and-whisker plots have the advantage of making no assumption about the underlying statistical distribution. The length of whiskers was set equal to 1.5 times the interquartile range (IQR), corresponding to 2.69 standard deviation (σ), i.e., about 99.3% of data in the case of a normal distribution.

#### 2.2.3. Multivariate Statistical Methods

Principal component analysis and cluster analysis are powerful statistical methods for characterising different behaviours among microclimate observations. Both statistical tools have demonstrated an effective capability of identifying sensor faults [25,26], classifying microclimate time series with very similar features [27,28,29], and optimising the number of sensors required for microclimate monitoring in museums [20,21].

*Principal Component Analysis.* Principal component analysis (hereafter called PCA) is a powerful multivariate method that explores the correlation structure between variables and depicts the similarities and dissimilarities between observations. Principal components are directions of maximum data variance obtained as linear combinations of the original variables. In this case, T and RH collected by each microclimate sensor are the variables, while observations are the set of hours with available measurements. The projections of observations (hours, in this case) over these directions maximising data variability are called scores. In contrast, the contributions of variables in the formation of a given component are called loadings, with *p*_[1]_ being the loadings in the formation of the first principal component (PC1); *p*_[2]_, the loadings of PC2; and so on. The scatterplot of the loadings corresponding to PC1 and PC2, which is commonly referred to as PC1/PC2 loading plot, was inspected visually, because it usually summarises the most relevant information from the data series. Loading plots corresponding to further components were also inspected, attempting to interpret the similarities between sensor nodes.

**Figure 4:**
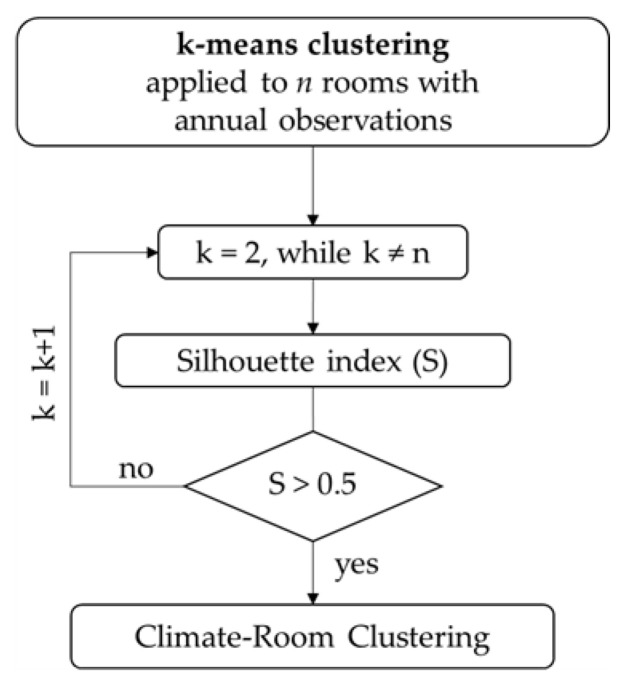
Schematic workflow of the k-means clustering.

*Cluster Analysis.* Cluster analysis (hereafter, CA) can be used to aggregate data in separate clusters. In the k-means clustering, data are assembled in different k clusters by an iterative algorithm so that the variance within each cluster (intra cluster) is minimised, while the variance between the clusters (inter cluster) is maximised [30]. The outputs for each cluster are the centroid and the Euclidean distance. The former is the mean value of individual variables (T or RH), whereas the latter identifies the distance of T and RH data of each room with respect to the centroid of its own cluster. The iterative approach adopted in this study is schematised in Figure 4.

Firstly, the Silhouette index (S) was used in order to identify a proper number of k clusters, as it allows us to objectively evaluate the quality of the clustering expressed as the within-cluster consistency of the i-th room (i.e., the cohesion of elements in a cluster) compared to other clusters (i.e., the separation of each cluster from others) [31]. *S* index ranges between −1 (i.e., i-th room does not belong to that cluster) and +1 (i.e., data are well clustered). Here, k was iteratively increased, starting with k = 2, in order to get the highest score of median S with a confidence level of 95% and taking S = 0.5 as the lower threshold. Nonetheless, when the median S coincides among two or more clustering, the optimal number of k was chosen by comparing the individual S score calculated for each room. As a rule of thumb, k should be lower than the number of rooms (*n*). Finally, the room located at the minimum Euclidean distance from the centroid was objectively taken as the most representative element for each cluster, which means being representative of the indoor climate-room cluster. The k-mean clustering was applied to the T and RH monthly averages. Such averages were calculated only when the number of days with hourly observations in a month was higher than 20 (i.e., more than 480 recordings) and the observations were equally distributed along the month. This was fundamental to avoid any biased interpretation of outcomes by applying the k-means clustering. For the application of CA, average monthly data were used to filter the daily fluctuations possibly related to specific episodes (e.g., higher number of visitors, museum-related activities) not attributable to the typical indoor climate conditions.

## 3. Results

This section is structured in three parts: Section 3.1 shows the outcomes of T and RH data pre-processing, while Section 3.2 and Section 3.3 show the results from the statistical methods applied to objectively identify similarities, if any, among rooms. Finally, Section 3.4 is devoted to the discussion of the a posteriori deployment of microclimate sensors based on the previous results.

### 3.1. Data Pre-Processing

**Figure 5:**
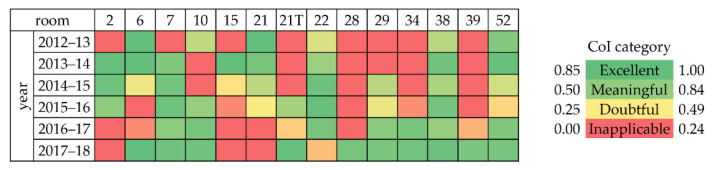
Results of the completeness index (CoI) for each room and each year over the period under study. Note that the term “year” here indicates the period (365 days) covering the months from June to May of the following year.

The CoI matrix (Figure 5) gives two types of information on the time series: the horizontal reading shows which microclimate sensors collected the most complete time series per year, while the vertical reading in column reveals which microclimate sensor collected the most complete time series over years. As an example, data collected in Room 7 were used to investigate the capability of artificial neural networks to predict short-term temperature evolution in museum environment [32]. Based on this matrix, for the aim of this research, the time frame selected was 1 June 2017–31 May 2018, because the quality of T and RH time series in this period was excellent, with CoI ranging between 0.88 and 1.00 in 10 out of 14 rooms (dark green boxes in Figure 5). The selected data subset comprised 12 months with available hourly observations, meaning that no failure in thermo-hygrometers occurred due to low batteries or missing record downloads. Room 22 (CoI = 0.34, yellow/orange box) as well as Rooms 2, 15, and 21 (CoI < 0.25, red boxes) did not comprise enough observations to conduct further statistical analyses.

### 3.2. Univariate Statistical Method

**Figure 6:**
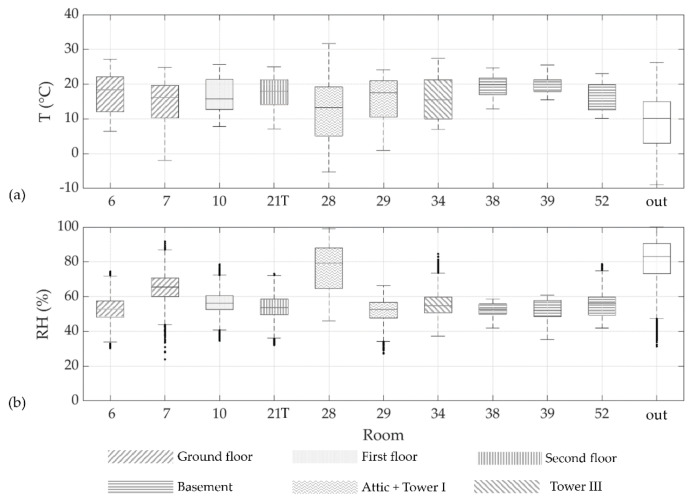
Box-and-whisker plots of temperature (**a**) and relative humidity (**b**) recorded in Rosenborg Castle over the period from 1 June 2017 until 31 May 2018. Additionally, outdoor air conditions retrieved from the ERA5 database [24] were also considered (labelled as “out”). The length of whiskers was set equal to 1.5 times the interquartile range (IQR).

Figure 6a,b shows the box-and-whisker plots of T and RH data, respectively, recorded in each room and outside (data retrieved from ERA5 database, labelled as “out”) over the selected period. Table 2 and Table 3 synthetically report the main statistical parameters for the interpretation of box-and-whisker plots. Outliers were visible only in RH plots (Figure 6b, black dots), although they were not excluded in the following analyses. In general, it can be noted that T boxes overlap in all rooms, whereas RH boxes behave differently among the rooms.

Box-and-whisker plots provide a synthetic visualisation of the hygrothermal behaviour, but this information does not provide a microclimate classification, except for adjoining locations such as Rooms 38 and 39 in the basement, which might be reasonably characterised by similar indoor climate conditions.

Moreover, Figure 6 illustrates a different performance for TRH28, which was deployed in a semi-confined attic where no artworks are exhibited or preserved (personal communication of the museum curator). In such location, observations collected by TRH28 were highly similar to the external conditions, also characterised by a higher IQR with respect to values collected in the other rooms.

### 3.3. Multivariate Statistical Methods

Room 28 was initially included in the following analysis. However, both Figure 6 and multivariate methods proved to be effective in identifying that the microclimate conditions measured in this room were not comparable with those of the other rooms, always revealing an isolated microclimate pattern.

*Principal Component Analysis*. PCA was carried out on the hourly T and RH data, separately. The main results are shown below.

**Figure 7:**
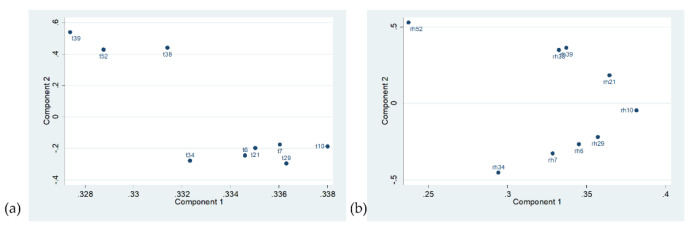
Scatter plot of the loadings corresponding to Component 2 versus Component 1 for the hourly data of (**a**) temperature and (**b**) relative humidity.

Regarding temperature, Table 4 shows that PC1 is the only component with an eigenvalue > 1, which explains 95% of the total data variance. Figure 7a shows the contribution of temperature sensors to PC1 and PC2 (loadings), which together explain 97.8% of the total data variability. PC1 loadings range between 0.328 and 0.338. This low variability could be ascribed to the high similarity in time evolution (i.e., parallel trajectories). Considering PC2 loadings, PCA seems to reveal two clusters of rooms based on the height with respect to the ground floor: a group established with Rooms 38, 39, and 52 (in the basement, with PC2 loadings ≥ 0.4) and another group containing all the rest (PC2 loadings ranging between −0.1 and −0.3).

In the case of RH, Table 4 reports that the two first components (PC1 and PC2) have an eigenvalue > 1 and explain 66.5% and 16.8% of the data variability, respectively. In Figure 7b, PC1 loadings range between 0.2 and 0.4, highlighting that the time evolution of RH observations is not markedly parallel in the rooms. PC2 loadings, instead, range between −0.5 and 0.5. Once again, on the basis of floor height, rooms in the basement form a cluster with some peculiarities: RH in Room 52 differs in terms of time evolution of relative humidity (PC2, see Figure 7b), whereas Rooms 38 and 39 appear extremely similar to each other. The other rooms seem to be characterised by a different microclimate pattern.

PCA highlights that one microclimate pattern is clearly attributable to rooms in the basement and another for Rooms 7 and 6 (ground floor), whereas the remaining rooms might be singularly grouped in other microclimate patterns.

**Figure 8:**
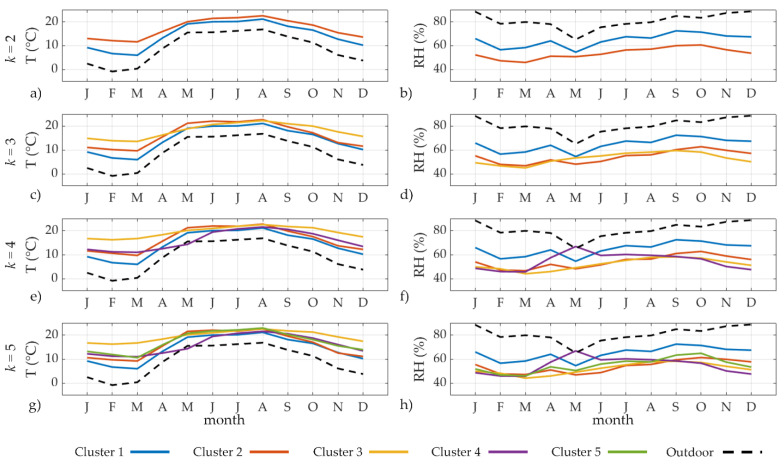
Time evolution of temperature (left panels) and relative humidity (right panels) in each cluster based on outcomes of different k-mean Cluster Analysis: (**a**,**b**) for k = 2; (**c**,**d**) for k = 3; (**e**,**f**) for k = 4; (**g**,**h**) for k = 5. Colour code: Cluster 1 in blue; Cluster 2 in orange; Cluster 3 in yellow; Cluster 4 in violet; Cluster 5 in green; outdoor conditions in black.

*Cluster Analysis.* Cluster analysis (CA) was applied to indoor monthly averages of T and RH observations following the workflow in Figure 4 and considering a number of clusters (k) from 2 to 5. Figure 8 shows the time evolution of temperature and relative humidity both indoors (based on the clustering k) and outdoors (extracted from the ERA5 database [24]).

Cluster 1 was characterised by the lowest monthly T (T = 6.0 °C) occurring in March (Figure 8a,c,e,g, blue lines) and the highest monthly RH (RH = 70.0%) occurring in September (Figure 8b,d,f,h, solid blue lines). Both indoor variables were highly correlated with the outdoor ones (dashed black lines). When k ≥ 3, Cluster 3 identified a microclimate pattern characterised by higher T and lower RH values compared the others, especially in winter months. Cluster 4 was characterised by a peculiar microclimate pattern recorded on average in May: it yields the lowest T (16.0 °C) and the highest RH (67.0%) in the castle (Figure 8e–h, solid violet lines). This microclimate behaviour was in contrast with outdoor conditions and those reflected by other clusters.

Regardless of the k value, Room 7 (cluster 1) is always characterised by a peculiar microclimate pattern (Cluster 1), showing that this room is the most sensitive to the infiltration of external air masses due to its proximity to an entrance door. Similarly, Rooms 38 and 39 are always included in the same cluster and, in case of k = 5, they are strongly in accordance with each other at S = 0.9 (cluster 3). The microclimate in these rooms is the least affected by the outdoor climate conditions due to the high thermal inertia related to the heavy masonries (wall thickness 1.5 m) compared to the other rooms (wall thickness 1.0 m): the average monthly T is equal to 19.4 °C, whereas RH = 52.0%.

Table 5 summarises the representative outcomes of the cluster analysis for k = 2, k = 3, k = 4 and k = 5, respectively. When k > 3, microclimate conditions in Room 52 are presented by a unique cluster with T = 15.9 °C and RH = 54.7% on average. Looking at the average TRH conditions for each k, it is evident that the clustering is temperature-driven, ranging between 14.4 °C (Cluster 1, Room 7, ground floor) and 19.4 °C (Cluster 3, k = 5, Rooms 38 and 39, basement).

According to the Silhouette index (S), k = 3 has the lowest median S score (S = 0.4, ranging between −0.2 and 1.0) whereas k = 2 yields the highest (S = 0.8 ranging between 0.5 and 1.0). A negative individual S score occurs only in Room 21T (S = −0.2 for k = 3, S = −0.1 for k = 4) meaning that, although similar, Room 21T is not well clustered with other rooms belonging to the same cluster. When k = 3, individual S scores of Cluster 3 range between −0.2 and 0.3, meaning that the similarity among TRH of these rooms is not highly significant, especially in Room 21T. This result seems reasonable, as Rooms 38, 39, and 52 are located in the basement, whereas Room 21T is on the second floor. Individual S scores of Cluster 2 in k = 5 range between 0.2 and 0.3, meaning that Rooms 29 and 34, located in the two towers, are not well clustered with Room 6 on the ground floor. Considering the S median, four clusters (k = 4) were found to be effective in the climate-room classification.

### 3.4. A-Posteriori Deployment of Microclimate Sensors

**Figure 9:**
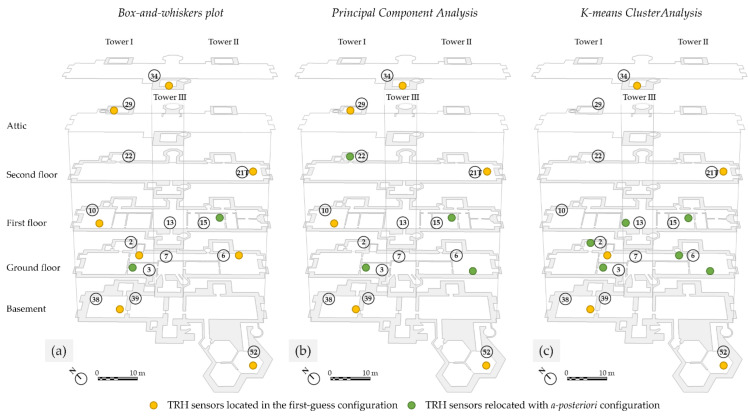
Plans of Rosenborg Castle. A posteriori deployment of the TRH sensors according to: (**a**) box-and-whisker plots, (**b**) principal component analysis, and (**c**) k-means cluster analysis.

The location of microclimate sensors according to the first-guess configuration, along with the position of climate-sensitive artworks selected by the museum conservators, are shown in Figure 2a,b. Based on the classifications derived from the three methods, three a posteriori deployments of microclimate sensors are shown in Figure 9a–c. For each method, the number of sensors to relocate was equal to the number of sensors that collected similar microclimate patterns, and, for this reason, may be moved to other positions. It is worth noticing that the relocation of these redundant sensors followed the priority provided by conservators, i.e., considering the climate-sensitivity of the selected artworks. In addition, the sensor in Room 28 (attic where no artworks are exhibited or preserved) was relocated in all the cases to Room 3. It has to be borne in mind that periodic calibration of sensors should be performed not only on a regular basis but also repeated every time a sensor is moved.

According to the climate-room classification resulting from the interpretation of box-and-whisker plots, it was found that Rooms 38 and 39 had a similar microclimate behaviour over the year. For this reason, the microclimate sensor originally located in Room 39 was moved to Room 15 (Figure 9a). This configuration would allow collection of microclimate data for two additional artworks.

From the principal component analysis, a similar microclimate behaviour was identified between Rooms 38 and 39, as well as between Rooms 6 and 7. For this reason, the microclimate sensors originally located in Rooms 39 and 7 could be moved to Rooms 15 and 3 (Figure 9b). This configuration would allow to collect microclimate data for four additional artworks.

From the k-means cluster analysis, four clusters were identified as representative of the four microclimate patterns in the Castle (Table 5). The sensors originally located in Rooms 6, 21T, 29, 34, and 39 could be relocated. The microclimate sensor in Room 6 was moved to the opposite side of the room, closer to the selected climate-sensitive artworks, in order to study whether microclimate conditions differed from the original sampling point in the proximity of artworks. The others were moved to Rooms 3, 13, and 15 (Figure 9c). This new configuration would allow to collect microclimate data for nine additional artworks.

To sum up, Table 6 summarises the results obtained by the three statistical approaches providing objectively the number of sensors that can be moved from the original location to another location close to artworks following the decision of conservators. Finally, the k-means cluster analysis has proven to be more sensitive to the intrinsic common characteristics of microclimate behaviour, identifying four microclimate patterns.

## 4. Conclusions

This paper applied three statistical methods for an a posteriori deployment of microclimate sensors in multi-room buildings in which valuable artworks are preserved. The proposed methodology is relevant because it could occur that in the first-guess configuration, microclimate sensors were deployed either in redundant microclimate sampling points (i.e., areas with similar microclimate behaviour) or far away from climate-sensitive artworks. For this reason, a rational approach would help conservators in relocating microclimate sensors to collect data at other sampling points, which might have been discarded due to budgetary limitations in the first-guess configuration. The approach was tested with data collected in several rooms of Rosenborg Castle (Copenhagen, Denmark) from 2012 until 2018.

One of the main differences with respect to similar studies previously reported is the use of box-and-whisker plots combined with multivariate statistical methods. The former are commonly used in the context of microclimate monitoring, as they allow a straightforward comparison of median values recorded by a set of sensors, as well as the interquartile range. Moreover, skewed distributions and extreme values are clearly visualized.

The main advantages in using box-and-whisker plots are that they can be easily interpreted by most users, and they can be obtained with many user-friendly statistical programs. On the other hand, T and RH variables can be analysed separately, so that their comparison does not consider the temporal evolution. For this reason, deployment of the sensors can be decided mainly on the basis of previous user’s experience, assessing the risks of inadequate microclimate conditions. In addition, the comparison among medians might not be sufficient to recognise differences in terms of statistical distribution. In this particular case, it turned out that RH values recorded by sensor in Room 28 and the one outdoors yielded a higher median and IQR compared with the rest. Conversely, they yielded the lowest median temperature in the period under study.

Interpreting the results of principal component analysis and cluster analysis is a complex task, as users need to have statistical skills and both require the use of advanced computer programs. In fact, several types of data pre-treatments can be applied in PCA, leading to different results [13,25]. However, these methods have the advantage of revealing hidden relationships between variables in a more overarching way than just looking at a single variable, and they are very powerful to discover stages (i.e., changes of trend) when long monitoring periods are considered. In this case, principal component analysis, separately applied to hourly temperature and relative humidity observations, identified several microclimate patterns: air conditions in the basement appeared distinctively different from the red, and two adjoining rooms on the ground floor yielded a similar microclimate. Cluster analysis, instead, applied to monthly average values of temperature and relative humidity, allowed us to identify four microclimate patterns: Room 7, basement (though room 52 was classified independently), and the rest.

The comparison of results using both PCA and CA is rarely used in reported studies about microclimate monitoring.

Some considerations regarding the limitations of our study are outlined here. Firstly, the application of multivariate statistics is advisable when several microclimate time series are available in the study, which is not always the case. Unfortunately, it was not possible to validate the proposed climate-room classification due to the lack of microclimate observations in the following years. Moreover, due to the limited availability of long-term measurements of other environmental variables, the proposed approach was tested only for the relocation of thermo-hygrometers. It would be advisable to integrate microclimate analysis with other environmental variables such as light, indoor air pollutants, and particulate matter to better design the configuration of sensors, even in view of conservation needs priority. Finally, the proposed a posteriori deployments have not been set out yet, which is planned to be carried out in the near future.

## Figures and Tables

**Table 1:** Technical features of the T and RH sensors (Tinytag View 2 TV-4050) used in the monitoring.

	Temperature	Relative Humidity
Sensor Type	Thermistor	Capacitive
Measurement range	−30 °C to 50 °C	0 to 100%
Uncertainty	0.4 °C	±3.0% RH at 25 °C

**Table 2:** Summary of the statistical parameters for temperature (°C) data over the period 1 June 2017, until 31 May 2018 (min = minimum; Q1 = first quartile; Q2 = second quartile or median; Q3 = third quartile; max = maximum; IQR = interquartile range).

Room	6	7	10	21T	28	29	34	38	39	52	Out
min	6.4	1.3	7.8	7.2	−5.2	0.9	7.0	12.8	15.5	10.1	−9.0
Q1	12.0	10.5	12.7	14.1	5.1	10.5	10.0	17.0	17.9	12.6	3.0
Q2	18.3	16.2	15.7	18.0	13.3	17.5	15.4	19.9	20.0	16.0	10.1
Q3	22.1	19.7	21.3	21.3	19.2	20.9	21.2	21.8	21.3	19.9	15.0
max	27.2	24.8	25.6	25.0	31.6	24.2	27.4	24.7	25.5	23.1	26.2
IQR	10.1	9.2	8.6	7.2	14.1	10.4	11.2	4.8	3.4	7.3	12.0

**Table 3:** Summary of the statistical parameters (same as in Table 2) for relative humidity (%) data over the period 1 June 2017, until 31 May 2018.

Room	6	7	10	21T	28	29	34	38	39	52	Out
min	30.3	23.8	34.7	32.0	46.1	27.1	37.3	41.8	35.4	41.9	31.3
Q1	48.1	60.2	52.6	49.5	64.8	47.7	50.6	49.9	48.5	49.4	73.2
Q2	53.0	65.7	56.3	53.6	79.4	52.7	54.9	52.9	52.1	56.3	83.2
Q3	57.5	70.8	60.5	58.6	88.0	56.7	59.8	55.8	57.7	59.8	90.5
max	74.4	91.7	78.5	73.2	98.9	66.4	84.6	58.8	60.8	78.7	100.0
IQR	9.5	10.6	7.9	9.1	23.2	9.1	9.2	6.0	9.3	10.3	17.3

**Table 4:** Summary of PCA outcomes obtained from hourly observations of temperature and RH (R2 is the proportion of variability explained by each component).

Component	Temperature		Relative Humidity	
	Eigenvalue	R^2^	Eigenvalue	R^2^
1	8.555	0.951	5.983	0.665
2	0.247	0.028	1.515	0.168
3	0.075	0.008	0.575	0.064
4	0.054	0.006	0.293	0.033
5	0.027	0.003	0.252	0.028
6	0.016	0.002	0.207	0.023
7	0.012	0.001	0.093	0.010

**Table 5:** Summary of k-mean cluster analysis (CA) applied to temperature (T) and relative humidity (RH) monthly data over the period from 1 June 2017 until 31 May 2018, in Rosenborg Castle. Table reports data on the cluster associated with each room; the individual Silhouette index (S) within the cluster; the median S over all clusters, and the centroid values for T and RH. The smallest distance from the centroid for each cluster is indicated by an asterisk (more than one asterisk is present for the same cluster in the case of equal smallest distances from the cluster centroid).

Rooms	Floor	k = 2	S	k = 3	S	k = 4	S	k = 5	S
6	ground	2	0.8	2	0.5	2	0.3	2	0.2
7	ground	1 *	1.0	1 *	1.0	1 *	1.0	1 *	1.0
10	first	2	0.7	2	0.4	2 *	0.5	5 *	0.5
21T	second	2 *	0.8	3	−0.2	2	−0.1	5 *	0.5
29	attic—tower I	2	0.8	2 *	0.6	2	0.4	2 *	0.3
34	tower III	2	0.5	2	0.7	2	0.6	2	0.2
38	basement	2	0.8	3 *	0.3	3 *	0.9	3 *	0.9
39	basement	2	0.8	3	0.3	3 *	0.9	3 *	0.9
52	basement	2	0.6	3	0.3	4 *	1.0	4 *	1.0
	Median Silhouette index (S):		**0.8**		**0.4**		**0.6**		**0.5**
		T (°C)	RH (%)	T (°C)	RH (%)	T (°C)	RH (%)	T (°C)	RH (%)
*Centroid Values*	Cluster 1	14.4	64.6	14.4	64.6	14.4	64.6	14.4	64.6
	Cluster 2	17.2	53.7	16.3	54.3	16.5	54.3	16.1	53.8
	Cluster 3			18.0	53.2	19.4	52.0	19.4	52.0
	Cluster 4					15.9	54.7	15.9	54.7
	Cluster 5							17.2	55.0

**Table 6:** Comparison among the results obtained by the three statistical methods. The number of sensors to move in the a posteriori deployment includes the identified redundant sensors and TRH28 placed in the first-guess configuration in the attic, where no artworks are exhibited or preserved.

	Box-and-Whisker Plots	Principal Component Analysis	k-Means Cluster Analysis
Number of identified microclimate patterns	8	7	4
Number of sensors to move	1	2	5

## Data Availability

Raw data were collected at Rosenborg Castle (Copenhagen, Denmark). Derived data supporting the findings of this study are available from the corresponding author F.F. on request.
